# Establishment and evaluation of a novel practical tool for the diagnosis of pre-sarcopenia in young people with diabetes mellitus

**DOI:** 10.1186/s12967-023-04261-w

**Published:** 2023-06-17

**Authors:** Ruina Li, Sixian Lin, Jiayuan Tu, Yongzhuang Chen, Bin Cheng, Xiaoqiao Mo, Tian Xie

**Affiliations:** 1 Department of Clinical Pharmacy, Union Shenzhen Hospital, Huazhong University of Science and Technology, Shenzhen, Guangdong China; 2 Neurosurgery Department, Kunming Medical University, Kunming, Yunnan China; 3 School of Nursing and Public Health, Yangzhou University, Yangzhou, Jiangsu China; 4 Yancheng No.1 People’s Hospital, Yancheng, China; 5 Department of Burns and Plastic Surgery, Union Shenzhen Hospital, Huazhong University of Science and Technology, Shenzhen, Guangdong China; 6 Department of Operating Room, Xinhua Hospital, Shanghai Jiao Tong University School of Medicine, Shanghai, China; 7 Department of General Surgery, Shanghai Ninth People’s Hospital, Shanghai Jiao Tong University School of Medicine, Shanghai, China

**Keywords:** Pre-sarcopenia, Diabetes mellitus, NHANES, Nomogram, Skeletal muscle mass

## Abstract

**Objective:**

Sarcopenia has been recognized as a third category of complications in people with diabetes. However, few studies focus on the reduction of skeletal muscle mass in young people with diabetes. The aim of this study was to investigate risk factors of pre-sarcopenia in young patients with diabetes and establish a practical tool to diagnose pre-sarcopenia in those people.

**Methods:**

Patients (*n* = 1246) enrolled from the National Health and Nutrition Examination Survey (NHANES) cycle year of 2011 to 2018 were randomly divided into the training set and validation set. The all-subsets regression analysis was used to select the risk factors of pre-sarcopenia. A nomogram model for the prediction of pre-sarcopenia in the diabetic population was established based on the risk factors. The model was evaluated by the area under the receiver operating characteristic curve for discrimination, calibration curves for calibration, and decision curve analysis curves for clinical utility.

**Results:**

In this study, gender, height, and waist circumference were elected as predictive factors for pre-sarcopenia. The nomogram model presented excellent discrimination in training and validation sets with areas under the curve of 0.907 and 0.912, respectively. The calibration curve illustrated excellent calibration, and the decision curve analysis showed a wide range of good clinical utility.

**Conclusions:**

This study develops a novel nomogram that integrates gender, height, and waist circumference and can be used to easily predict pre-sarcopenia in diabetics. The novel screen tool is accurate, specific, and low-cost, highlighting its potential value in clinical application.

## Introduction

Diabetes mellitus (DM) is a metabolic disorder characterized by hyperglycemia resulting from insulin deficiency, insulin resistance, or both. It has become a major global health epidemic with high incidence worldwide. The global prevalence rate of diabetes has been projected to be 8.8% (415 million individuals) in 2015 and this number is supposed to increase to 10.4% (642 million individuals) by 2040 [1]. Previous studies have shown that diabetes is associated with dysfunction, damage, and failure of various organs, especially the blood vessels, heart, kidneys, eyes, and nerves. Among these, sarcopenia—an age-related reduction in muscle mass and muscle function condition, has been described as a noteworthy complication in the elderly with diabetes. Since insulin resistance, and hyperglycemia are associated with the acceleration of the rate of muscle loss in the addition of diabetes [2, 3].

Sarcopenia, first described by Irving Rosenberg in 1988, is an age-related loss of muscle mass and function disorders, which brings enormous clinical challenge, and burdens of economic and daily life for individual [2]. Sarcopenia affects more than 5% of the elderly aged 60 to 70 years, and 11–50% of those aged 80 or above [4]. The European Working Group on Sarcopenia in Older People (EGWSOP) first defined “sarcopenia” as low skeletal muscle strength with low skeletal muscle mass or decline in physical performance and defined “pre-sarcopenia” as low skeletal muscle mass [5]. Sarcopenia and low skeletal muscle mass in the elderly have been shown to have a higher risk of falls, frailty, disability, and mortality [6, 7]. Studies have shown that diabetes is nowadays a generally acknowledged risk factor for sarcopenia, and the overall prevalence of sarcopenia is higher in diabetics, ranging from 7 to 29% [8–10]. It’s of great importance to take preventive strategies in people with a higher risk of sarcopenia the sooner the better since studies have suggested that sarcopenia is a progressive disorder that is strongly related to age [10, 11]. Consequently, focus should not only be paid on the prevention and treatment of sarcopenia in the elderly with diabetes but also the early screening methods for risk factors of “pre-sarcopenia” in young or mid-life diabetics.

It has been demonstrated that sarcopenia, an age-related process, is associated with frailty and the microvascular and macrovascular risks of diabetes, which are emerging as severe complications leading to disability and mortality [12]. Studies have indicated that sarcopenia can promote the development of diabetes in the elderly and vice versa. Results from other studies suggested that insulin resistance, chronic hyperglycemia may explain the high prevalence of sarcopenia in diabetes [9, 13]. Sarcopenia obesity (SO), the co-existence of obesity and low muscle mass/function, has been observed as a complication in diabetes with sarcopenia. The higher prevalence SO in diabetes may be explained by different mechanisms, such as aging, change in hormone, lack of exercise, and other factors that have impacts on body composition changes, especially in muscle mass reduction and increase in fat mass [14]. Obviously, the increased prevalence of sarcopenia has a heavy impact on the quality of life and physical activity in diabetic elderly population.

Recent studies suggest that sarcopenia is a potentially reversible or preventable condition, which means diabetic patients with sarcopenia are assumed to benefit from early diagnosis and early intervention, especially in the early stage of sarcopenia [15]. However, the determination of skeletal muscle mass is extremely limited, and requires professional equipment, such as dual-energy X-ray absorptiometry (DXA), bio-impedance analysis (BIA), X-ray computed tomography (CT), or magnetic resonance imaging (MRI).

Considering the impact on the quality of life for people with diabetes, much more attention has been paid to the identification and prevention of sarcopenia in diabetic individuals [16]. However, the practical and available method for the diagnosis of sarcopenia is still lacking, and insufficient methods are available for pre-sarcopenia, especially for diabetes people with pre-sarcopenia. Here we established a novel practical tool for the diagnosis of pre-sarcopenia in people with diabetes. Patients with laboratory-confirmed diabetes in the National Health and Nutritional Examination Surveys (NHANES) were studied, and the classification of low skeletal muscle mass was based on the criteria designated by “Foundation for the National Institutes of Health (FNIH) Sarcopenia Project” [17].

## Methods

### Data source and study population

In this study, data were obtained from NHANES, which is conducted by the National Center for Health Statistics (NCHS), part of the Centers for Disease Control and Prevention (CDC). In brief, this survey was based on US adults with complete dual-energy X-ray absorptiometry results (*n* = 17,927) in NHANES data (cycle 2011–2018). Furthermore, people with diabetes were included, which can be classified based on 1 of the following criteria: glycohemoglobin (HbA1C) ≥ 6.5% (48 mmol/mol), the value of fasting plasma glucose (FPG) ≥ 126 mg/dL (7.0 mmol/L), 2-h plasma glucose (2-h PG) ≥ 200 mg/dL (11.1 mmol/L) after a 75-g oral glucose tolerance test (OGTT), or being told to take lower-glucose medicine by the doctor in the questionnaire [18].

### Measurements and definition of pre-sarcopenia

In the NHANES, body composition was measured by DXA using the Hologic QDR-4500 A fanbeam densitometer (Hologic, Inc., Bedford, MA, USA). Exclusion criteria: participants with pregnant or weight over 450 lb (204 kg) or 6 ft, 5 in. (195 cm) or participants with missing data of variables (such as Waist circumference, OGTT etc.). Inclusion criteria: participants were 18 years of age or older, and with data of DXA and without missing variables. To obtain complete results of DXA data, data files of DXA in NHANES from 2011 to 2018 were collected. Appendicular skeletal muscle mass (ASM), the sum of lean mass for both arms and legs, is usually adopted in clinical practice. We performed analysis using skeletal muscle mass index (SMI) calculated as ASM adjusted for body mass index which was recommended by the Foundation for the National Institutes of Health (FNIH) Sarcopenia [19, 20]. Man with SMI < 0.789, or woman < 0.512 was recognized as having low muscle mass, defined as pre-sarcopenia [16].

### Variable estimation

Information on gender, age, race, standing height, weight, body mass index (BMI), waist circumference, blood pressure, complete blood count, and standard biochemistry was collected from the demographics and examination public release files released by NHANES. The weight, standing height, and waist circumference of participants were obtained from the body measure examinations in a standardized way. Race is divided into four categories: Mexican American; non-Hispanic white; non-Hispanic black, and others. BMI is defined as body weight (kilograms) divided by height (meters) squared. Hypertension is identified by systolic blood pressure measurements > 140 mmHg or diastolic blood pressure measurements > 90 mmHg from the blood pressure examination released by NHANES.

### Model establishment and validation

Patients were randomly divided into a training set and a validation set at a ratio of 7:3, respectively. An all-subsets regression method was used to investigate the useful combination of factors that could most precisely predict pre-sarcopenia in diabetes [21]. Accessible variables were analyzed by all-subset analysis to select potential predictive factors. Calibration curves were subsequently drawn to assess the goodness-of-fit between the nomogram-predicted probability and the actual proportion. The sensitivity, specificity, and optimal cutoff of model performance were evaluated through the area under the receiver operating characteristic curve (AUC). In essence, a wider separation in the curves indicates better discrimination. In addition, decision curve analysis (DCA) was conducted to determine whether our established nomogram was suitable for clinical utility by estimating the net benefits at different threshold probabilities [22]. All statistical analysis was performed using the R statistical software (R Foundation for Statistical Computing, Vienna, Austria), and the nomogram was constructed using the *leaps* package [23].

### Statistical analysis

All statistical analyzes were performed with SPSS 26.0 software. Continuous variables were presented as means and standard deviation and analyzed using the Mann–Whitney U test or the student’s *t*-test. The Chi-square test and Fisher’s exact test were used in the comparison of categorical variables, the results of which were expressed as numbers (percentages). All *P* values were two-tailed, and *P* < 0.05 was considered statistically significant.

## Results

### Baseline characteristics

**Fig. 1:**
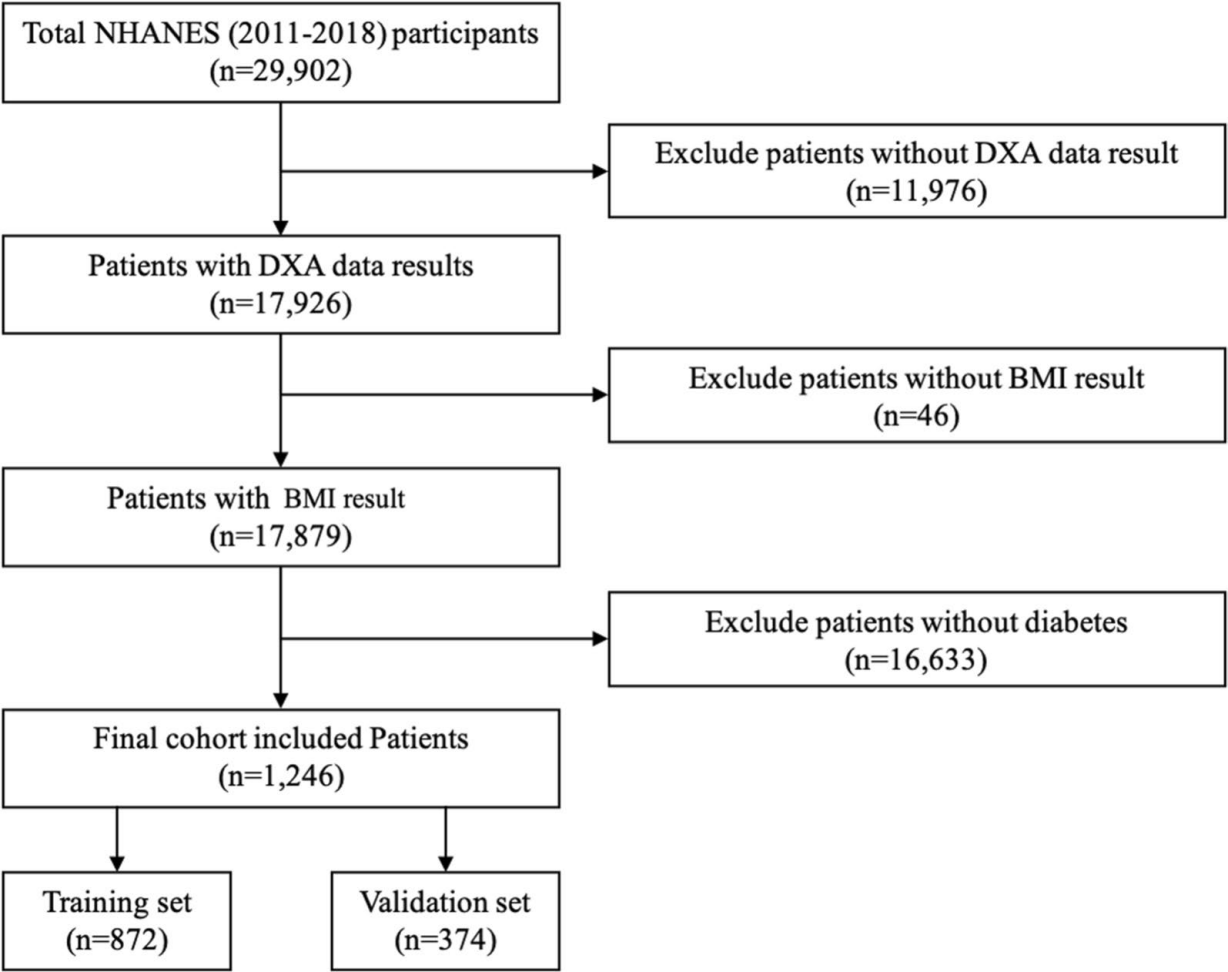
Schematic of specific patient screening process. The flowchart of how the present sample of participants was composed. NHANES, National Health and Nutrition Examination Survey. *DXA* Dual energy x-ray absorptiometry. *BMI* body mass index

A total of 1246 participants were included in this study. A flowchart of the study selection methodology is shown in Fig. 1. The total prevalence of pre-sarcopenia in participants with diabetes was 18.86% (*n* = 235) in our study. The average age of pre-sarcopenia participants (47.5 ± 11.4 years) was significantly older than non-pre-sarcopenia participants (45.8 ± 10.6 years). Male participants accounted for 51% of non-pre-sarcopenia participants and 46% of pre-sarcopenia, while female participants accounted for 49% of non-pre-sarcopenia patients and 54% of pre-sarcopenia. Pre-sarcopenia participants have significantly higher BMI (35.8 ± 8.51 Vs. 32.4 ± 7.34 kg/m2), shorter height (159 ± 9.74 Vs. 168 ± 9.45 cm), and higher waist circumference (113 ± 18.5 Vs. 108 ± 16.8 cm) than those without pre-sarcopenia (all *P* < 0.05). The detailed baseline of other characteristics was listed in Table 1.

**Table 1:** Baseline characteristics of participants according to Sarcopenia Status in US adults, NHANES 2011–2018

Characteristic	Non pre-sarcopenia	Pre-Sarcopenia	*P* value
Participants (*n*)	1011	235	
Gender			0.183
Male (%)	516 (51.0%)	108 (46.0%)	
Female (%)	495 (49.0%)	127 (54.0%)	
Age (years)	45.8 (10.6)	47.5 (11.4)	0.033
Race (%)			< 0.001
Mexican American	164 (16.2%)	78 (33.2%)	
Non-Hispanic Black	300 (29.7%)	16 (6.81%)	
Non-Hispanic White	254 (25.1%)	62 (26.4%)	
Other	293 (29.0%)	79 (33.6%)	
BMI (kg/m^2^)	32.4 (7.34)	35.8 (8.51)	< 0.001
Height (cm)	168 (9.45)	159 (9.74)	< 0.001
Waist circumference (cm)	108 (16.8)	113 (18.5)	< 0.001
Weight (kg)	92.1 (23.5)	91.7 (27.0)	0.816
Hypertension (Yes, %)	251 (24.8%)	72 (30.6%)	0.080
Diastolic pressure (mmHg)	75.8 (16.0)	74.4 (17.3)	0.281
Systolic pressure (mmHg)	126 (25.5)	127 (27.1)	0.664
White blood cell count (1000 cells/µL)	7.85 (2.40)	8.25 (2.06)	0.011
Lymphocyte (1000 cells/µL)	2.38 (0.92)	2.42 (0.74)	0.430
Monocyte (%)	0.57 (0.19)	0.58 (0.18)	0.630
Neutrophils (%)	4.64 (1.85)	4.97 (1.68)	0.008
Eosinophils (%)	0.21 (0.18)	0.23 (0.20)	0.228
Basophils (%)	0.06 (0.05)	0.06 (0.06)	0.647
Red blood cell (million cells/µL)	4.81 (0.51)	4.88 (0.56)	0.073
Hemoglobin (g/dL)	14.0 (1.66)	14.3 (1.60)	0.009
Hematocrit (%)	41.7 (4.44)	42.6 (4.45)	0.005
Mean cell volume (fL)	87.0 (6.43)	87.6 (5.83)	0.141
Mean cell hemoglobin (pg)	29.2 (2.69)	29.5 (2.40)	0.208
Mean cell hemoglobin concentration (g/dL)	33.6 (1.18)	33.6 (0.98)	0.774
Mean platelet volume (fL)	8.46 (0.94)	8.66 (1.03)	0.007
Red cell distribution width (%)	13.8 (1.44)	13.8 (1.42)	0.733
Platelet (1000 cells/µL)	251 (70.9)	249 (74.7)	0.777
Albumin (g/L)	41.6 (3.69)	41.2 (3.37)	0.119
Globulin (g/L)	30.6 (5.18)	31.2 (4.09)	0.060
Protein, total (g/L)	72.2 (5.09)	72.4 (4.18)	0.543
Alanine aminotransferase (U/L)	30.6 (24.9)	34.1 (22.7)	0.046
Aspartate aminotransferase (U/L)	28.1 (31.7)	29.4 (18.4)	0.397
Alkaline phosphatase (U/L)	77.2 (27.9)	87.3 (36.2)	< 0.001
Gamma-glutamyl transferase (U/L)	40.7 (54.8)	48.7 (64.7)	0.091
Bilirubin, total (µmol/L)	9.86 (5.16)	9.54 (5.10)	0.395
Creatinine (µmol/L)	79.8 (69.9)	68.1 (19.7)	< 0.001
Blood urea nitrogen (mg/dL)	13.5 (6.46)	13.7 (5.03)	0.496
Creatine Phosphokinase (IU/L)	170 (182)	137 (225)	0.048
Lactate dehydrogenase (U/L)	134 (35.5)	140 (40.0)	0.079
Calcium (mmol/L)	2.34 (0.10)	2.34 (0.10)	0.516
Iron (µmol/L)	14.1 (6.48)	14.2 (5.88)	0.747
Phosphorus (mmol/L)	1.20 (0.19)	1.19 (0.19)	0.758
Uric acid (µmol/L)	328 (88.7)	322 (89.2)	0.439
25OHD2 + 25OHD3 (nmol/L)	58.9 (25.6)	58.8 (23.9)	0.927
25OHD2 (nmol/L)	5.59 (16.1)	4.38 (10.6)	0.16
25OHD3 (nmol/L)	53.4 (25.0)	54.4 (24.2)	0.557
Sodium (mmol/L)	139 (2.70)	138 (2.69)	0.464
Potassium (mmol/L)	3.99 (0.36)	4.02 (0.37)	0.239
Chloride (mmol/L)	102 (3.30)	102 (3.51)	0.009
Osmolality (mOsm/kg)	280 (5.69)	280 (5.49)	0.334
Glycohemoglobin (%)	7.55 (2.12)	7.77 (2.04)	0.151
Fasting Glucose (mmol/L)	8.90 (3.85)	9.81 (4.38)	0.037
Two-hour oral glucose tolerance (OGTT) (mmol/L)	12.6 (5.22)	15.4 (6.88)	0.022
Insulin (µU/mL)	23.8 (39.2)	33.2 (66.5)	0.151
Triglyceride (mmol/L)	1.92 (2.55)	2.31 (2.35)	0.109
HDL (mg/dL)	46.3 (13.6)	46.5 (13.7)	0.842
Total cholesterol (mmol/L)	5.04 (1.38)	5.18 (1.38)	0.161
LDL (mmol/L)	3.00 (1.04)	3.06 (1.01)	0.597

*BMI* Body Mass Index, *HDL* High density lipoprotein, *LDL* Low density lipoprotein

### Nomogram construction

**Fig. 2:**
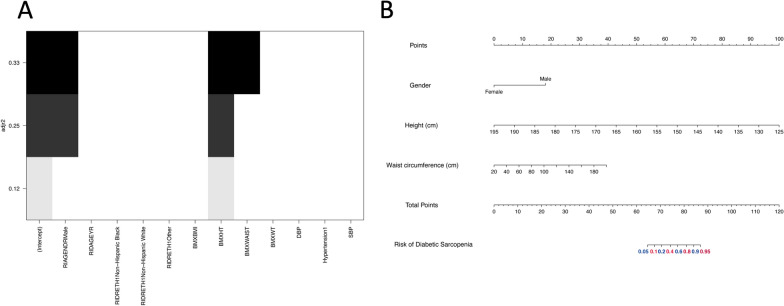
Nomogram for the diagnosis of pre-sarcopenia. **A** All-subset regression analysis. The importance of variates was calculated as adjusted r-square value. The optimal model owns the highest adjusted r-square value. Y-axis presented the adjusted r-square value, and X-axis presented the potential predictive factors. The adjusted r-square value was calculated as the sum of colored variate, and higher adjusted r-square value means the better prediction. In this study, the adjusted r-square value of race and height was 0.25, waist circumference, race and height were 0.33. **B** Predictive nomogram model for pre-sarcopenia in young diabetic people. The nomogram model was based on gender, height, and waist circumference. And each predictive factor has a scoring point, the total score points of those three factors may indicate the risk of pre-sarcopenia in young diabetic people

Participants were randomly divided into the training set (*n* = 872) and the validation set (*n* = 374) by stratified random sampling at a ratio of 7:3. The training set was used to develop the nomogram model while the validation set was used as the evaluation of the performance of the predictive model. In order to establish a practical model, only demographic parameters were selected as potential predictive factors such as age, gender, race, BMI, height, weight, systolic blood pressure, diastolic blood pressure, waist circumference and hypertension. Then, an all-subset regression analysis was performed to identify the best predictive factors. The adjusted R square showed that the combination of gender, height and waist circumference made the highest R square of 0.33 (Fig. 2A), which means those three factors were the predictive factors for the pre-sarcopenia in patients with diabetes. Next, a nomogram based on gender, height and waist circumference was established (Fig. 2B). Noticeably, people with male gender, shorter height and higher waist circumference were at higher risk of pre-sarcopenia. For example, a diabetic man with 170 cm height and 120 cm waist circumference may have a total score of 76 which means he may have the possibility of 50% for the risk of pre-sarcopenia.

### Performance and validation of the nomogram

**Fig. 3:**
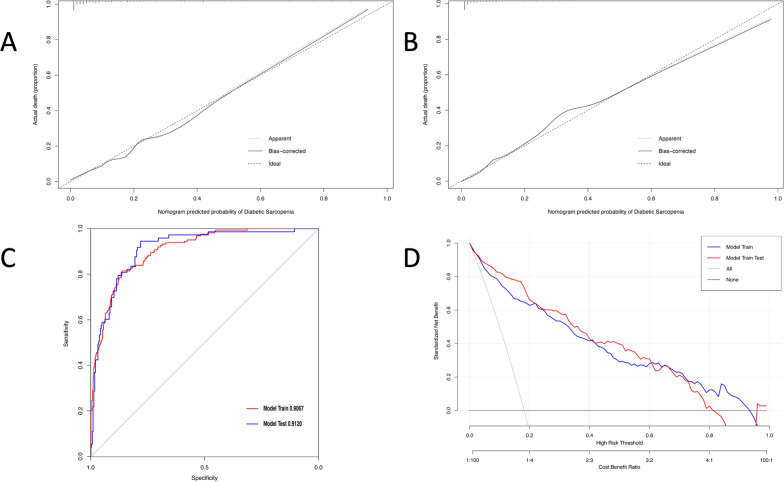
Performance and validation of the nomogram model. **A** Calibration curve of the nomogram for pre-sarcopenia in the training set. **B** Calibration curve of the nomogram for pre-sarcopenia in the validation set. The X-axis represents the predicted probability and the Y-axis denotes the actual proportion. The diagonal dotted line indicates the best prediction by an ideal model. The apparent line represents the uncorrected performance of the nomogram while the solid line shows the bias-corrected performance. **C** Receiver operating characteristic curve of the nomogram. Performance evaluation of the nomogram in the training and validation sets. The area under the receiver operating characteristic curve (AUC) in the training set was 0.907. In the validation set, the AUC was 0.912. **D** Decision curve analysis. The net benefit curves for the nomogram model are shown. X-axis indicates the threshold probability for pre-sarcopenia risk and Y-axis indicates the net benefit. The red and blue line represents the improvement predictive nomogram in the training and validating set. The gray line represents the assumption that all patients used the nomogram model. The black line represents the assumption that no patients use the nomogram model to predict the risk of pre-sarcopenia. Our study showed a wide threshold for the net benefit in training and validating sets

To evaluate the performance of our nomogram model, the calibration curves were conducted in both the training set and the validation set. Both calibration curves showed good fit between the actual observed values and the predicted values with the range of 0 to 1.0, indicating a robust calibration of the nomogram model (Fig. 3A, B). The discrimination was assessed by the receiver operation curves (ROC). Our nomogram model showed excellent discrimination in both training and validation sets with AUCs of 0.907 and 0.912, respectively (Fig. 3C). In addition, the decision curve analysis (DCA) was conducted to evaluate the clinical use. DCA curves showed that the net benefit probability was between 0% and 82% and 0% and 95% in the training set and the validation set, respectively, which means our model could bring more benefits for the prediction of pre-sarcopenia in diabetes (Fig. 3D).

## Discussions

Sarcopenia, characterized by a degenerative loss of skeletal muscle strength, mass, and progressive age-related function, remains an under-recognized problem in clinical practice, especially in diabetes mellitus [24]. Stratified by gender and age, the prevalence rate of sarcopenia in patients with type 2 diabetes mellitus (T2DM) ranges widely from 10 to 40% [25]. A cross-sectional analysis in NHANE III demonstrated that each 10% increase in skeletal muscle index (SMI) was accompanied by a 12% relative decrease in prediabetes prevalence (95% CI 1–21%), after adjusting for multiple confounding factors [26]. Early diagnosis and early intervention are of great importance to the prevention, and treatment of sarcopenia in diabetes. In our study, we have developed a novel, practical, predictive nomogram based on three independent factors-gender, height, and waist circumference, which may help clinicians predict the risk of developing sarcopenia in patients with diabetes mellitus. The nomogram demonstrated good discriminative, calibration capabilities, and clinical utility, and it suggested that males with short height and higher waist circumference may have a higher risk of diabetic sarcopenia.

The impact of gender on the incidence of sarcopenia in diabetes patients is controversial. The Health ABC Study performed on 2675 patients with whole body dual-energy X-ray absorptiometry demonstrated that the diabetic patients had greater loss of appendicular lean mass compared with nondiabetic older adults, and men have greater muscle mass loss than women in diabetic groups, which is consistent with previous study [27, 28]. Similarly, the English Longitudinal Study of Aging (*n* = 3404) found that older adults with diabetes have a higher risk of sarcopenia in men (OR 2.43, 95% CI 1.5–3.9) but no significance in women (OR 1.49, 95% CI 0.83–2.68) [29], of which is consistent with our study that men may have a higher risk of sarcopenia compared with women in diabetics. However, several studies demonstrated that female has a higher prevalence compared with that in men, especially in Asia population [30, 31]. It has been demonstrated that Asian has higher body fat percentages and lower BMI compared to Caucasians [32]. The higher risk of pre-sarcopenia in female patients with diabetes is probably associated with more muscle mass decline and more fat mass increase during aging, and higher changes in hormonal [14]. After the menopausal transition, the concentrations of estrogens and androgens decrease dramatically in women [33], while the decline of sex steroids is much slower in men [34]. The relationship between race, the level of sex steroids and sarcopenia needs to be further investigated.

Another finding of our study was that height was a predictive factor for pre-sarcopenia in diabetes patients. Han et al. reported that males in the lowest tertile group (< 174.8 cm) of height showed a 4.4-fold increased risk for type 2 diabetes mellitus compared to those in the highest tertile group (≥ 181.5 cm) [35]. In addition, Ji et al. found that Individuals with greater height loss (OR 2.30, 95% CI  1.26–4.42 with ratio of height change below − 2%) were frailer, more likely to be diagnosed with sarcopenia regardless of age and sex [36]. Our nomogram model demonstrated that height was an important risk factor in the current study, which coincides with previous studies.

An interesting finding from our result is that pre-sarcopenia was associated with a higher waist circumference. Korea National Health and Nutrition Examination Survey (KNHANES), a cross-sectional study, which enrolled 6832 Korean adult participants found that the prevalence of sarcopenia in participants with large waist circumference was about sixfold greater than those with normal waist circumference (11.5% vs. 1.9%) [37]. Another cross-sectional study including 656 elderly inpatients demonstrated that frailty in the elderly was characterized by large waist circumference, low skeletal muscle mass, and high body fat mass, the symptom of which was described as “sarcopenic obesity” [38]. Moreover, a study has found in the case of similar BMI, male subjects with diabetes had decreased lean body mass and increased body fat mass compared with non-diabetics [39]. The pathogenesis of sarcopenic obesity is complex, with the possible interplay between aging, sex-specific hormonal changes, pro-inflammatory cytokines induce, and physical inactivity. The results suggested that sarcopenia has a strong association with higher waist circumference, which was consistent with our results.

Nevertheless, there are several limitations to this study. First, we used cross-sectional data in our study, which came from an observational survey, and cannot determine causality but only the association. However, it has the capacity to assess the prevalence of pre-sarcopenia in diabetes and provide a risk probability for pre-sarcopenia in diabetes. Additionally, hypoglycemic drugs should also be taken into account, since evidence from epidemiological studies indicates that men with T2DM using insulin sensitizers, such as metformin and thiazolidinediones, lost significantly less skeletal muscle mass (− 1.1% vs. −2.9%) or appendicular skeletal muscle mass (− 1.8% vs. − 4.4%) than those treated without insulin sensitizers [40]. In our study, the percentage of pre-sarcopenia in diabetes with insulin sensitizers was lower than those with other anti-diabetic medication (15.85% vs. 18.86%, *P* = 0.499), yet there was no statistical difference because of the small sample size of insulin-sensitizers subgroup (*n* = 82). The prediction efficacy of our nomogram model for diabetic patients should be further validated and confirmed by external validation and large, multicenter prospective studies.

In conclusion, the study has identified the predictors of pre-sarcopenia in the diabetes population. Diabetic people with short height and higher waist circumference may have a higher risk of pre-sarcopenia, especially men. In our study, we established a practical predictive nomogram model based on those three predictors to identify the high risk of pre-sarcopenia diabetic people. Our study may provide a convenient and useful tool for physicians and diabetic people to prevent sarcopenia at risk, which may ameliorate the disability and mortality in diabetic people and promote their quality of life.

## Data Availability

Data can be downloaded from the cdc.gov website.
